# Findings From Severe Maternal Morbidity Surveillance and Review in Maryland

**DOI:** 10.1001/jamanetworkopen.2022.44077

**Published:** 2022-11-29

**Authors:** Carrie Wolfson, Jiage Qian, Pamela Chin, Cathy Downey, Katie Jo Mattingly, Kimberly Jones-Beatty, Joanne Olaku, Sadaf Qureshi, Jane Rhule, Danielle Silldorff, Robert Atlas, Anne Banfield, Clark T. Johnson, Donna Neale, Jeanne S. Sheffield, David Silverman, Kacie McLaughlin, Güneş Koru, Andreea A. Creanga

**Affiliations:** 1 Department of International Health, Johns Hopkins Bloomberg School of Public Health, Baltimore, Maryland; 2 Department of Population, Family and Reproductive Health, Johns Hopkins Bloomberg School of Public Health, Baltimore, Maryland; 3 Mercy Medical Center, Baltimore, Maryland; 4 Howard County General Hospital, Columbia, Maryland; 5 MedStar St Mary’s Hospital, Leonardtown, Maryland; 6 Department of Gynecology and Obstetrics, Johns Hopkins School of Medicine, Baltimore, Maryland; 7 Sinai Hospital of Baltimore, Baltimore, Maryland; 8 Luminis Health Anne Arundel Medical Center, Annapolis, Maryland; 9 Independent researcher; 10 Department of Obstetrics and Gynecology, George Washington School of Medicine and Health Sciences, Washington, DC; 11 Maternal and Child Health Bureau, Health Resources and Services Administration, Rockville, Maryland; 12 Department of Health Policy and Management, University of Arkansas for Medical Sciences, Fayetteville

## Abstract

**Importance:**

In the US, more than 50 000 women experience severe maternal morbidity (SMM) each year, and the SMM rate more than doubled during the past 25 years. In response, professional organizations called for birthing facilities to routinely identify and review SMM events and identify prevention opportunities.

**Objective:**

To examine SMM levels, primary causes, and factors associated with the preventability of SMM using Maryland’s SMM surveillance and review program.

**Design, Setting, and Participants:**

This cross-sectional study included pregnant and postpartum patients at 42 days or less after delivery who were hospitalized at 1 of 6 birthing hospitals in Maryland between August 1, 2020, and November 30, 2021. Hospital-based SMM surveillance was conducted through a detailed review of medical records.

**Exposures:**

Hospitalization during pregnancy or within 42 days post partum.

**Main Outcomes and Measures:**

The main outcomes were admission to an intensive care unit, having at least 4 U of red blood cells transfused, and/or having COVID-19 infection requiring inpatient hospital care.

**Results:**

A total of 192 SMM events were identified and reviewed. Patients with SMM had a mean [SD] age of 31 [6.49] years; 9 [4.7%] were Asian, 27 [14.1%] were Hispanic, 83 [43.2%] were non-Hispanic Black, and 68 [35.4%] were non-Hispanic White. Obstetric hemorrhage was the leading primary cause of SMM (83 [43.2%]), followed by COVID-19 infection (57 [29.7%]) and hypertensive disorders of pregnancy (17 [8.9%]). The SMM rate was highest among Hispanic patients (154.9 per 10 000 deliveries), primarily driven by COVID-19 infection. The rate of SMM among non-Hispanic Black patients was nearly 50% higher than for non-Hispanic White patients (119.9 vs 65.7 per 10 000 deliveries). The SMM outcome assessed could have been prevented in 61 events (31.8%). Clinician-level factors and interventions in the antepartum period were most frequently cited as potentially altering the SMM outcome. Practices that were performed well most often pertained to hospitals’ readiness and adequate response to managing pregnancy complications. Recommendations for care improvement focused mainly on timely recognition and rapid response to such.

**Conclusions and Relevance:**

The findings of this cross-sectional study, which used hospital-based SMM surveillance and review beyond the mere exploration of administrative data, offers opportunities for identifying valuable quality improvement strategies to reduce SMM. Immediate strategies to reduce SMM in Maryland should target its most common causes and address factors associated with preventability identified at individual hospitals.

## Key Points

**Question:**

What are the severe maternal morbidity (SMM) levels, primary causes, and factors associated with its preventability in birthing hospitals in Maryland?

**Findings:**

This cross-sectional study of hospital-based SMM surveillance in Maryland identified 192 SMM events, with obstetric hemorrhage (43%), followed by severe COVID-19 infection (30%) and hypertensive disorders of pregnancy (9%), being the most common causes. Nearly two-thirds of SMM events reviewed were deemed preventable, with changes in clinician-level factors and interventions in the antepartum period having the largest potential to alter the SMM outcome.

**Meaning:**

Immediate strategies to reduce SMM in Maryland should target its most common causes and address factors associated with SMM preventability identified at individual hospitals.

## Teaser

This cross-sectional study of hospitalized pregnant and postpartum patients in Maryland examines causes and factors associated with severe maternal morbidity.

## Introduction

More than 50 000 women experience severe maternal morbidity (SMM) annually in the US. Moreover, the SMM rate more than doubled during the past 25 years and is 2 times higher for non-Hispanic Black than non-Hispanic White women.^1^ The Centers for Disease Control and Prevention (CDC) defines SMM as potentially life-threatening conditions or complications resulting from labor and delivery that can significantly affect a woman's health.^2^ Severe maternal morbidity events, which are 100 times more prevalent than maternal mortality, can be considered near-misses for maternal deaths.^3^ Reviews of SMM events can provide more learning opportunities than reviews of maternal deaths alone. The reduction of preventable SMM may also stem increasing maternal mortality rates because they share similar risk factors.^4^

Prior examination of SMM using mainly administrative hospital data^5^ demonstrated that approximately half of adverse maternal outcomes in the US are attributable to preventable harm or unintended consequences from clinical practice and system of delivering perinatal care.^6,7^ The CDC, American College of Obstetricians and Gynecologists (ACOG), and Society for Maternal-Fetal Medicine (SMFM) recommend that birthing facilities routinely identify and review SMM events.^3,8,9^ Reviewing SMM allows for characterization of circumstances leading to SMM and determination of whether SMM was preventable. By identifying potentially preventable SMM and associated factors, facilities can recommend and implement specific practice changes or quality improvement initiatives to prevent future adverse outcomes.

The case definition for hospital-based SMM identification proposed by ACOG/SMFM includes admission to an intensive care unit (ICU) and/or transfusion of 4 U or more of blood.^3^ This 2-factor criterion identified a significant number of SMM events and offered critical learning opportunities for clinicians and hospitals in prior studies.^10,11,12^

Until 2020, the only data on SMM in Maryland were from administrative hospital discharge databases.^7^ Such data, primarily collected for billing purposes, are prone to coding errors and lack clinical nuance needed for real-time, in-depth reviews to inform SMM prevention efforts.^5,9,13^ In July 2020, the Maryland Maternal Health Innovation Program (MDMOM) piloted an SMM surveillance and review program working with 6 of the 32 birthing hospitals in Maryland, covering approximately one-quarter of the more than 60 000 births in the state annually. This initiative is a component of a series of interventions implemented in 2020 to reduce maternal mortality in Maryland. The surveillance and review program examined factors that contribute to SMM and identified prevention strategies through the systematic and comprehensive review process recommended by ACOG/SMFM. This study examines SMM levels, primary causes, factors associated with its preventability, and recommendations for care improvement.

## Methods

The SMM surveillance and review program in Maryland was designed to identify and review life-threatening conditions in pregnant and postpartum patients admitted to participating hospitals. The SMM definition was adapted from ACOG/SMFM’s proposal for hospital-based surveillance during pregnancy or within 42 days post partum: patients admitted to an ICU or critical care unit (CCU) and/or with 4 U or more of red blood cells (RBCs) transfused and/or affected by emerging public health threats during the year that required hospital care (eFigure 1 in the Supplement). During the pilot study, a confirmed COVID-19 infection that required inpatient hospital care met the case definition. The institutional review board at the Johns Hopkins Bloomberg School of Public Health deemed the study exempt from review because it does not qualify as human subjects research as defined by the US Department of Health and Human Services; therefore, patient informed consent was not required. This cross-sectional study followed the Strengthening the Reporting of Observational Studies in Epidemiology (STROBE) reporting guideline.

Severe maternal morbidity events were identified as close to real time as possible, typically within 1 month, by trained nurse or physician assistant abstractors (P.C., C.D., K.J.M., K.J.-B., J.O., S.Q., J.R., and D.S.). Collected data included structured elements, summary case narratives with a timeline of key events, and unstructured information on preventability and recommendations from each event. Abstractors reviewed the electronic health record and any other maternal and newborn records (eg, birth certificate) to document information about the patient, including race and ethnicity, and SMM event using a standardized electronic form developed for the pilot. Racial and ethnic categories were defined by the hospitals’ electronic health software and were specified in the abstraction form as Asian, Black or African American, American Indian or Alaska Native, Native Hawaiian or other Pacific Islander, White, other (specify), and unknown. Ethnicity was specified as Hispanic or Latina, not Hispanic or Latina, and unknown. Each event was reviewed by a hospital-based perinatal review committee, typically consisting of a lead obstetrician, quality improvement specialist(s), and data abstractor(s). The committee determined the primary (ie, underlying) cause of morbidity and contributing conditions through review of the abstracted information and case narrative. The abstraction form provided checkboxes for the top morbidity causes and an “other” open-ended field for rare causes. The committee collaboratively used a standardized guide adapted from the model of preventability proposed by Geller et al^14^ to assess whether the event was preventable, note factors that influenced the outcome, and identify opportunities for improvement. Events were considered preventable if a change to 1 or more condition(s) or situation(s) related to the clinician, system, or patient during the antepartum, intrapartum, and/or postpartum period could have prevented the SMM event or made the outcome less severe. Review committees also identified practices that were performed well and made recommendations for care improvement.

Data are from the pilot phase of Maryland’s SMM surveillance and review program conducted over 16 months (August 1, 2020, to November 30, 2021) in 6 birthing hospitals. Hospitals were selected to represent a range in maternity care levels (ie, 1 level IV, 4 level III, and 1 level I hospital), delivery volume, and geographic spread, including urban and rural locations, and comprised more than 25% of births in the state. All invited hospitals elected to participate, and data on all events within participating hospitals that met the SMM case definition were abstracted and reviewed (N = 192).

### Statistical Analysis

The MDMOM program researchers (C.W. and J.Q.) cleaned and analyzed case data using Stata software, version 15 (StataCorp LLC). Rates of SMM were calculated overall and by race and ethnicity per 10 000 deliveries in pilot hospitals in 2019 from the Agency for Health Care and Quality State Inpatient Databases; rates were compared using 2-tailed, unpaired *t* tests.^15,16^ When data were available, using χ^2^ tests, we compared characteristics of patients with SMM and their delivery to those who had live births in Maryland during 2020; also, fetal deaths in patients with SMM were compared against the corresponding 2019 Maryland rate. A 2-sided *P* < .01 was considered statistically significant. Birth data were obtained from CDC WONDER (Wide-ranging Online Data for Epidemiologic Research).^17^ Using univariate analyses, we assessed levels, primary causes, timing, preventability (overall and by race and ethnicity), and patient, clinician, and health system factors associated with SMM. The SMM rates and preventability were stratified by race and ethnicity because of the large racial and ethnic disparities that are well documented in adverse maternal health outcomes. Analyses were conducted for the full sample and excluding COVID-19 infection cases (for comparison with prior SMM research). Excluded cases were those in which COVID-19 infection was the primary cause of morbidity or that met the SMM criteria because of COVID-19 only (n = 58).

Data collected via text fields (eg, preventability factors, recommendations, and practices performed well) were analyzed using content analysis techniques.^18^ Recommendations were coded according to the 5Rs framework proposed by ACOG’s Alliance for Innovation on Maternal Health for patient safety bundles and commonly used for maternity care quality improvement initiatives: readiness, recognition and prevention, response, reporting/system learning, and respectful, equitable, and supportive care.^19,20,21^ Two independent researchers (C.W. and J.Q.) analyzed text-field data to reach consensus.

## Results

### Patient Characteristics

Across the 6 hospitals, 192 SMM events were identified and reviewed. Patients with SMM had a mean (SD) age of 31 (6.49) years; 9 (4.7%) were Asian, 27 (14.1%) were Hispanic, 83 (43.2%) were non-Hispanic Black, and 68 (35.4%) were non-Hispanic White. More than half of the SMM events involved ICU/CCU admission (107 [55.7%]), 92 (47.9%) involved transfusion of 4 U or more of RBCs, and 60 (31.3%) involved severe COVID-19 infections. Some events had overlapping criteria: 39 (20.3%) involved both ICU/CCU admission and blood transfusion, 24 (12.5%) involved ICU/CCU admission and severe COVID-19 infection, and 2 (1.0%) involved all 3 criteria. The most common timing was ante partum (83 [43.2%]), followed by post partum within 8 hours of delivery (54 [28.1%]) (eFigure 2 in the Supplement). Of antepartum or intrapartum SMM (n = 109), approximately one-quarter occurred in patients at 20 to 27 weeks’ gestation (n = 26) and one-quarter in patients at 37 weeks’ gestation or later (n = 27) (eFigure 3 in the Supplement). When COVID-19 cases were excluded (eFigure 2 in the Supplement), the most common timing of SMM was post partum within 8 hours of delivery (53 [39.6%]). Among this group with antepartum or intrapartum SMM, 35 cases (63.6%) occurred at 34 weeks’ gestation or later.

**Figure 1.**
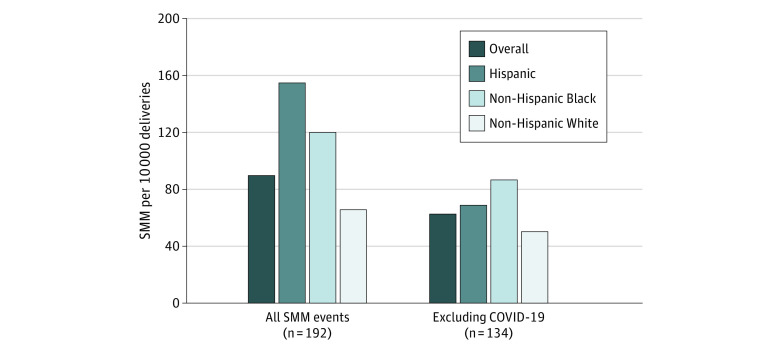
Severe Maternal Morbidity (SMM) Rates by Race and Ethnicity Data are from the Maryland SMM Surveillance and Review Database; denominators are based on 2019 deliveries in pilot hospitals.

The SMM rate was highest among Hispanic patients (154.9 per 10 000 deliveries), mainly driven by COVID-19 infections (Figure 1). The rate for non-Hispanic Black patients was nearly 50% higher than for non-Hispanic White patients (119.9 vs 65.7). Exclusion of COVID-19 events reduced the rate to 62.7 per 10 000 deliveries. Differences between non-Hispanic Black and White patients were significant with and without COVID-19 cases. Compared with the 2020 Maryland live-birth cohort, patients with SMM were more often non-Hispanic Black (43.2% vs 30.5%).

Patients in our series differed significantly from the full 2020 live-birth cohort in Maryland in all measured patient and delivery characteristics (Table 1). Notably, patients with SMM were older and more likely to be uninsured. Higher proportions of patients with SMM who experienced a live birth (n = 138) delivered infants who were preterm (58 of 128 [45.3%] vs 6941 of 68 554 [10.1%]), had low birth weight (41 of 127 [32.3%] vs 5792 of 68 554 [8.4%]), and were admitted to the neonatal ICU (54 of 126 [42.9%] vs 5540 of 68 554 [8.1%]). In addition, patients with SMM had pregnancies that resulted in stillbirth more frequently than among all births in Maryland during 2019 (10 of 138 [7.2%] vs 466 of 69 020 [0.7%]).

**Table 1.** Characteristics and Delivery Outcomes Among Patients With SMM Events (August 1, 2020, to November 30, 2021) and Live Births (January 1 to December 31, 2020) in Maryland^a^

Characteristic	All SMM (N = 192)	Excluding COVID-19 (n = 134)	Statewide births (n = 68 554)
Maternal age, y			
<20	5 (2.6)	5 (3.7)	2469 (3.6)
20-24	26 (13.5)	18 (13.4)	9414 (13.7)
25-29	37 (19.3)	24 (17.9)	17 628 (25.7)
30-34	63 (32.8)	43 (32.1)	22 596 (33.0)
35-39	41 (21.4)	29 (21.6)	13 234 (19.3)
≥40	20 (10.4)	15 (11.2)	3213 (4.7)
Maternal race and ethnicity^b^			
Asian	9 (4.7)	6 (4.5)	4603 (6.7)
Hispanic	27 (14.1)	12 (9.0)	13 034 (19.0)
Non-Hispanic			
Black	83 (43.2)	60 (44.8)	20 937 (30.5)
White	68 (35.4)	52 (38.8)	28 120 (41.0)
Other or unknown^c^	5 (2.6)	4 (2.9)	1860 (2.7)
Insurance type			
Private	103 (53.7)	72 (53.7)	38 998 (56.9)
Public	75 (39.1)	54 (13.3)	27 044 (39.4)
Self-pay or no insurance	14 (7.3)	8 (6.0)	2188 (3.2)
Prior births			
0	49 (25.5)	34 (25.4)	26 040 (38.0)
1	50 (26.0)	30 (22.4)	22 897 (33.4)
2	49 (25.5)	34 (25.4)	11 732 (17.1)
3	22 (11.5)	19 (7.0)	4684 (6.8)
≥4	22 (11.5)	17 (12.7)	3201 (4.7)
Timing of prenatal care initiation^d^			
First trimester	128 (66.7)	90 (67.2)	49 581 (72.3)
Second trimester or later	32 (16.7)	26 (19.4)	15 480 (22.6)
No prenatal care	5 (2.6)	4 (3.0)	929 (1.4)
Significant medical history	145 (75.5)	108 (80.6)	NA
Obesity	74 (38.5)	49 (36.6)	19 054 (27.8)
Mental health disorder	58 (30.2)	46 (34.3)	NA
Asthma	37 (19.3)	25 (18.7)	NA
Chronic hypertension	34 (17.7)	23 (17.2)	2557 (3.7)
Substance use	29 (15.1)	25 (18.7)	NA
Anemia	26 (13.5)	21 (15.7)	NA
Sexually transmitted infection	17 (8.9)	13 (9.7)	NA
Diabetes	15 (7.8)	11 (8.2)	750 (1.1)
Cardiovascular conditions	11 (5.7)	8 (6.0)	NA
Complications in current pregnancy	108 (56.3)	83 (61.9)	NA
HDP	20 (10.4)	18 (13.4)	6026 (8.8)
Placental abnormality	19 (9.9)	18 (13.4)	NA
Anemia	13 (6.8)	10 (7.5)	NA
Complications in prior pregnancy^e^	81 (55.1)	59 (58.4)	NA
Fetal death or stillbirth	70 (47.6)	47 (46.5)	NA
HDP	19 (12.9)	14 (13.9)	NA
Delivery during hospitalization with SMM event	138 (71.9)	122 (91.0)	NA
Delivery mode^f^			
Vaginal delivery	40 (29.0)	34 (27.9)	45 427 (66.3)
Spontaneous	36 (90.0)	31 (91.2)	43 615 (96.0)
Assisted	4 (10.0)	3 (8.8)	1812 (4.0)
Cesarean delivery^g^	98 (71.0)	88 (72.1)	23 114 (33.7)
Planned	35 (61.2)	32 (36.4)	NA
Emergency	60 (35.7)	53 (43.4)	NA
Live birth^f^^,^^h^	128 (92.8)	113 (92.6)	NA
PTB (<37 wk gestation)	58 (45.3)	50 (44.3)	6941 (10.1)
Early (<32 wk)	15 (11.7)	13 (11.5)	1150 (1.7)
Moderate (32-33 wk)	9 (7.0)	6 (5.3)	797 (1.2)
Late (34-36 wk)	34 (26.6)	31 (27.4)	4994 (7.3)
LBW (<2500 g)	41 (32.3)	34 (30.4)	5792 (8.4)
NICU admission	54 (42.9)	47 (41.6)	5540 (8.1)
Fetal death or stillbirth^f^^,^^i^	10 (7.3)	9 (7.4)	NA
Gestational age, mean (range)			
Weeks	31 (24-39)	33 (24-39)	NA
Days	2 (2-2)	6 (2-2)	NA

Abbreviations: HDP, hypertensive disorders of pregnancy; LBW, low birth weight; NA, not available; NICU, neonatal intensive care unit; PTB, preterm birth; SMM, severe maternal morbidity.

^a^ Data are from the Maryland SMM Surveillance and Review Database and the natality (2016-2020, expanded) and fetal deaths (2014-2019, expanded) records of the Centers for Disease Control and Prevention WONDER (Wide-ranging Online Data for Epidemiologic Research) database. The SMM events include patients during pregnancy or within 42 days post partum who are admitted to an intensive care unit or critical care unit and/or with 4 U or more of red blood cells transfused and/or admitted to a hospital for treatment of COVID-19 infection. All *P* values assessing differences in group distributions of all SMM vs statewide births are statistically significant at a 2-sided *P* < .01, and all characteristics were compared using χ^2^ analyses for which there was corresponding statewide data.

^b^ Race and ethnicity for statewide deliveries are from the birth certificate, which is self-reported.

^c^ Other includes American Indian or Alaska Native and Native Hawaiian or other Pacific Islander.

^d^ Timing of prenatal care missing for 27 patients with SMM (14.1%) and 2564 live births (3.7%).

^e^ Calculated from SMM events in which patients had a prior pregnancy (n = 147).

^f^ Calculated from SMM events that occurred during the delivery hospitalization (n = 138).

^g^ Cesarean delivery type missing for 3 cesarean deliveries.

^h^ Preterm, low birth weight, and NICU admission for SMM patients calculated out of live-birth deliveries with non-missing values for relevant characteristics (birth weight missing for 1 and NICU status missing for 2 live-birth deliveries).

^i^ Stillbirth data for statewide cohort are based on 2019 fetal deaths, and the percentage is the number of stillbirths in 2019 per all 2019 births (live birth and stillbirth combined).

Among the 192 patients with SMM, more than three-quarters had a significant medical history, including obesity (74 [38.5%]), a mental health disorder (58 [30.2%]), asthma (37 [19.3%]), and chronic hypertension (34 [17.7%]). More than half had a complication in the current pregnancy, most commonly a hypertensive disorder of pregnancy (HDP) (20 [10.4%]), placental abnormality (19 [9.9%]), or anemia (13 [6.8%]). Nearly half of patients with SMM (70 of 147 [47.6%]) who had been previously pregnant experienced a fetal death or stillbirth in 1 or more previous pregnancies.

### Primary Cause of SMM and Blood Loss Detail

Obstetric hemorrhage was the most frequent primary cause of SMM (83 [43.2%]), followed by severe COVID-19 infection (57 [29.7%]), HDP (17 [8.9%]), cardiovascular conditions (11 [5.7%]), and non–COVID-19 infections (10 [5.2%]) (Table 2). Obstetric hemorrhage was the most common cause of SMM among non-Hispanic Black (33 [39.8%]) and non-Hispanic White patients (35 [51.5%]), followed by COVID-19 infection (22 [26.5%] for non-Hispanic Black patients and 16 [23.5%] for non-Hispanic White patients) and HDP (10 [12.0%] for non-Hispanic Black patients and 4 [5.9%] for non-Hispanic White patients).

**Table 2.** Primary Cause of SMM Events and Distribution Overall and Excluding COVID-19 Events^a^

Primary cause of SMM	No. (%) of patients	
	All SMM (N = 192)	Excluding COVID-19 (n = 134)
Obstetric hemorrhage	83 (43.2)	82 (61.2)
COVID-19 infection	57 (29.7)	NA
Hypertensive disorders of pregnancy	17 (8.9)	17 (12.7)
Cardiovascular condition	11 (5.7)	11 (8.2)
Infection (non–COVID-19)	10 (5.2)	10 (7.4)
Hematologic^b^	3 (1.6)	3 (2.2)
Asthma	2 (1.0)	2 (1.5)
Neurologic conditions^c^	2 (1.0)	2 (1.5)
Pulmonary embolism	2 (1.0)	2 (1.5)
Other^d^	5 (2.6)	5 (3.7)

Abbreviations: NA, not applicable; SMM, severe maternal morbidity.

^a^ Data are from the Maryland SMM Surveillance and Review Database.

^b^ Sickle cell anemia (n = 1), iron deficiency anemia (n = 1), and blood clotting disorder (n = 1).

^c^ Seizure disorder (n = 1) and stroke (n = 1).

^d^ Anaphylaxis (n = 1), brain tumor (n = 1), cervical cancer (n = 1), motor vehicle injury (n = 1), and type 1 diabetes (n = 1).

The mean (SD) quantitative blood loss among 101 patients with abnormal blood loss was 3360.1 (2131.9) mL (eTable 1 in the Supplement). These patients received a mean of 9.4 U of blood products (range, 1-46 units; 6 patients received <4 U of RBCs but met other SMM inclusion criteria). A massive transfusion protocol was initiated for 34 of 101 SMM events with abnormal blood loss (all received ≥4 U of RBCs).

### SMM Preventability, Practices Performed Well, and Recommendations

**Figure 2.**
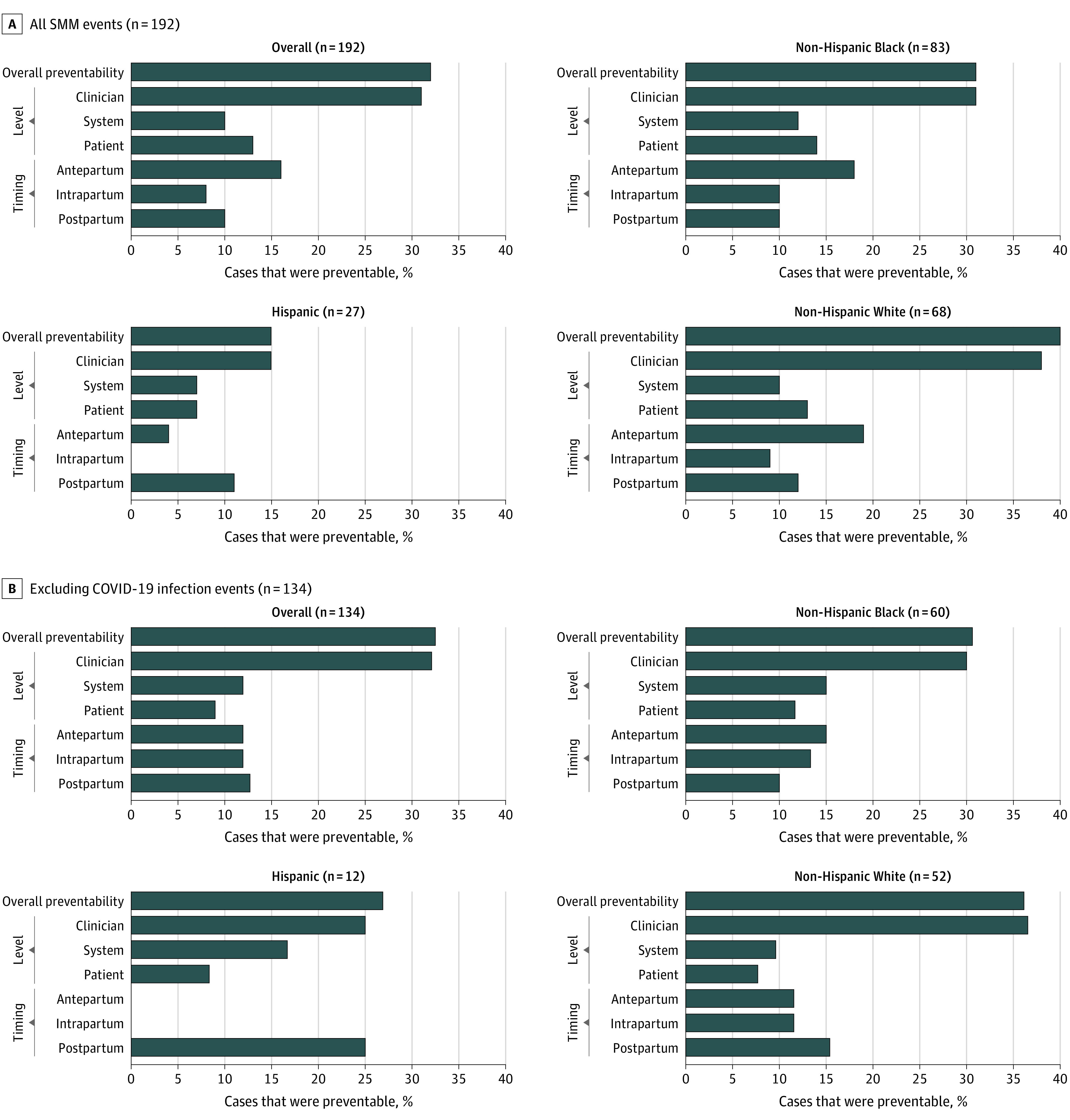
Preventability and Factors That Could Have Altered the Severe Maternal Morbidity (SMM) Outcome by Race and Ethnicity Multiple factors could be provided by data abstractors for each case. The SMM events can have factors that could have altered the outcome at multiple levels and timings. Data are from the Maryland SMM Surveillance and Review Database.

Hospital review committees determined that nearly one-third (61 [31.8%]) of SMM events were preventable with changes to clinician, system, and/or patient factors (without COVID-19 cases, the preventability rate was similar at 32.8%) (Figure 2). Clinician-level factors had the potential to alter the outcome in 60 of the 61 SMM events deemed preventable (31.3% of overall events), system-level factors in 19 events (9.9% overall), and patient-level factors in 24 events (12.5% overall). Changes in the antepartum period were identified as having the highest chance to alter the SMM outcome (31 [16.1%] of overall events). By race and ethnicity, 4 events (14.8%) among Hispanic patients were deemed preventable, 26 (31.3%) among non-Hispanic Black, and 27 (39.7%) among non-Hispanic White patients. Without COVID-19 cases, 3 cases (25.0%) were preventable among Hispanic patients, 18 (30.0%) among non-Hispanic Black patients, and 20 (38.5%) among non-Hispanic White patients. Clinician-, system-, and patient-level factors were noted as contributing factors at similar rates among non-Hispanic Black and White patients, but clinician factors were noted at lower rates among Hispanic patients.

**Figure 3.**
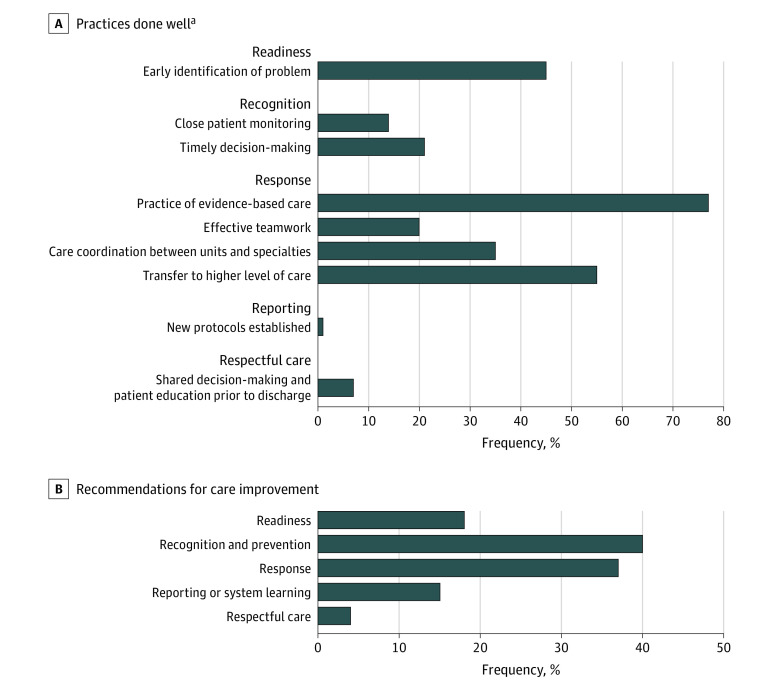
Practices Performed Well and Recommendations for Care Improvement Using the 5Rs Framework Noted Among the 192 Severe Maternal Morbidity (SMM) Events The 5Rs are readiness, recognition and prevention, response, reporting/system learning, and respectful, equitable, and supportive care. Multiple practices and recommendations were allowed and could be provided by data abstractors for each case. Data shown are absolute numbers of SMM events. Data are from the Maryland SMM Surveillance and Review Database. ^a^Fields for capturing this information were open-ended and unprompted; not mentioning these practices for a particular event does not mean they did not occur.

Through qualitative analyses, 9 groups of effective practices were identified and grouped by the 5Rs framework domains (Figure 3A). Review committees noted the practice of evidence-based care (response domain) in 77 events (40.1%), transfer to a higher level (response domain) in 55 events (28.6%), and early identification of the problem (readiness domain) in 45 events (23.4%). Most recommendations from this SMM series related to recognition and prevention as well as response (Figure 3B). Specific recommendations related to recognition included timely assessment, screening for and diagnosis of pregnancy complications, enhancing vital sign monitoring during hospitalization, and follow-up on abnormal tests (eTable 2 in the Supplement). Response recommendations included timely initiation of treatment for patients with severe range blood pressure values and abnormal bleeding, implementation of surgical care per clinical guidance, strengthening teamwork and communication within labor and delivery units, timely engagement with specialized care, coordination of care within and across hospital systems, and warm handoff of patients.

## Discussion

Maryland’s SMM surveillance and review program is the first to apply the method proposed by ACOG/SMFM to all SMM events identified in a state-level project. Obstetric hemorrhage was the main cause of SMM, followed by severe COVID-19 infection and HDP. Findings regarding non–COVID-19 primary causes of SMM are like those reported in the few prior studies^22,23^ applying similar criteria for SMM identification. The high proportion of patients with COVID-19 infection is noteworthy; COVID-19 infection is associated with other pregnancy complications, such as HDP, postpartum hemorrhage, and other infections.^24^ In our data, nearly half of patients with severe COVID-19 infection were either admitted to an ICU or received a blood transfusion. Of note, 54 SMM events (28.1%) occurred during nondelivery hospitalizations, a level that is higher than previously reported,^25^ and contributed to by COVID-19.

Patients with SMM were more likely to be 35 years or older and non-Hispanic Black, lack insurance coverage, and have obesity compared with the Maryland cohort with live births. These findings are similar to those reported in Illinois’s SMM surveillance with similar criteria for event identification^22^ and in a sample of patients with SMM across the US identified using hospital discharge data.^1^ Our data account for 25.1% of all births and 29.5% of SMM cases in Maryland based on hospital discharge data. These characteristics are also more common among patients with maternal deaths.^26^ In addition, more than half of patients with SMM in our series had a complication during the index pregnancy, and more than half with a previous pregnancy experienced pregnancy complications in a previous pregnancy. This finding speaks to the importance of recognizing and closely monitoring high-risk obstetric patients.

As expected, characteristics and delivery outcomes are similar for patients with SMM in our surveillance and those with pregnancy-associated or pregnancy-related deaths in Maryland in recent years (2010-2018).^27^ Notably, delayed or no prenatal care accompanied approximately 15.0% to 20.0% of SMM events in the present study and pregnancy-associated deaths according to prior research; a mental health diagnosis was present in 30.2% of patients with SMM and 34.7% among those with pregnancy-associated deaths; rates of fetal death were 7.3% among patients with SMM and 6.7% among pregnancy-related deaths.^20^ There were also important differences: hemorrhage was much more common among individuals with SMM (43.2% vs 15.0%). Severe maternal morbidity was observed more frequently during the antepartum period, whereas pregnancy-related deaths were more frequent post partum. These differences stem, in part, from the surveillance definition, identifying SMM within 42 days and mortality up to 1 year after pregnancy, and highlight the merit of surveillance and review of both SMM and maternal deaths—and go beyond the mere identification of adverse maternal events in administrative hospital data.

In our study, nearly two-thirds of SMM events were preventable. Clinician factors contributed to 31.3% of SMM events, system factors to 9.9%, and patient factors to 12.5%. Clinician factors were noted in nearly every event that was deemed preventable. Other facility-based SMM review projects have reported similar rates of preventability and an even higher contribution of clinician factors.^22,23,28^ In tandem with previous studies reporting that clinician-level factors are associated with more severe SMM outcomes,^6^ quality improvement initiatives should focus on clinician-level interventions for maximum impact. In addition, in our series, recommendations for care improvement were most often focused on the recognition and prevention and response domains within the 5Rs framework.

Of importance, non-Hispanic Black and Hispanic patients had disproportionately high rates of SMM compared with non-Hispanic White patients, but a higher proportion of SMM events in non-Hispanic White patients were deemed preventable. This finding may be due to differences in the proportion of cause of SMM by race and ethnicity, with non-Hispanic White patients experiencing the highest proportion of obstetric hemorrhage. Quality improvement initiatives that address racial disparities in SMM and focus on interventions that target preventable outcomes can reduce disparities and the incidence of adverse outcomes overall.^29^

The current study demonstrates the value of hospital-based surveillance of SMM and the feasibility of the standardized SMM surveillance method proposed by ACOG/SMFM. Hospital-based surveillance identifies fewer false-positive cases than administrative hospital discharge data and provides more nuanced information to identify strategies for prevention.^10^ Data from the 6 hospitals participating in the pilot appear to be representative of the state of Maryland. Through sensitivity analysis using statewide hospital discharge data and *International Statistical Classification of Diseases and Related Health Problems, Tenth Revision* codes, we compared the primary cause of morbidity in the pilot hospitals vs the 26 hospitals that did not participate; no significant difference was found in the distribution of primary morbidity causes (eTable 3 in the Supplement). Our data account for 25.1% of all births and 29.5% of SMM cases in Maryland based on hospital discharge data. The higher proportion of SMM events in pilot hospitals was expected because level III and IV hospitals are overrepresented in our program.

### Limitations

This study has some limitations. Despite using a standardized surveillance definition and data abstraction form, reports and overall assessment of SMM event preventability are subject to differential misclassification across hospitals because each has its own review committee. Contemporaneous data on patients without SMM were not available for comparison with patients with SMM, and the 2020 live-birth cohort used for comparison excludes non–live-birth pregnancy outcomes. In addition, our findings may not be generalizable to other states.

## Conclusions

Hospital-based SMM surveillance and review offered important opportunities for identifying impactful quality improvement strategies to reduce the burden of SMM. Immediate strategies to reduce SMM in Maryland should target its most common causes and address factors associated with SMM preventability identified at individual hospitals. On the basis of findings from the pilot program, the MDMOM program is developing new initiatives to reduce SMM in Maryland and has expanded the SMM surveillance and review in June 2022 to include 20 hospitals, covering nearly three-quarters of births in the state. This program can be used as a model by other states interested in learning how to best prevent SMM.

## Notes

Supplement. **eFigure 1.** Severe Maternal Morbidity Surveillance Definition **eFigure 2.** Timing of Severe Maternal Morbidity Events **eFigure 3.** Gestational Age at the Time of Antepartum and Intrapartum SMM Events **eTable 1.** Blood Loss and Transfusion in Patients With SMM by Primary Cause **eTable 2.** Condensed Recommendations for Care Improvement Organized by the 5Rs **eTable 3.** Distribution of Primary Cause of Severe Maternal Morbidity in Surveillance and Non-Surveillance Hospitals, 2019
