# Supplementary material for: Findings From Severe Maternal Morbidity Surveillance and Review in Maryland
Source: JAMA Netw Open. 2022 Nov 29;5(11):e2244077. doi: 10.1001/jamanetworkopen.2022.44077 (PMC9709651; doi:10.1001/jamanetworkopen.2022.44077)
Supplement: Supplement. — eFigure 1. Severe Maternal Morbidity Surveillance Definition eFigure 2. Timing of Severe Maternal Morbidity Events eFigure 3. Gestational Age at the Time of Antepartum and Intrapartum SMM Events eTable 1. Blood Loss and Transfusion in Patients With SMM by Primary Cause eTable 2. Condensed Recommendations for Care Improvement Organized by the 5Rs eTable 3. Distribution of Primary Cause of Severe Maternal Morbidity in Surveillance and Non-Surveillance Hospitals, 2019 [file jamanetwopen-e2244077-s001.pdf]

Supplementary Online Content

Wolfson C, Qian J, Chin P, et al. Findings from severe maternal morbidity surveillance and review in Maryland. *JAMA Netw Open*. 2022;5(11):e2244077. doi:10.1001/jamanetworkopen.2022.44077

eFigure 1. Severe Maternal Morbidity Surveillance Definition
eFigure 2. Timing of Severe Maternal Morbidity Events
eFigure 3. Gestational Age at the Time of Antepartum and Intrapartum SMM Events
eTable 1. Blood Loss and Transfusion in Patients With SMM by Primary Cause
eTable 2. Condensed Recommendations for Care Improvement Organized by the 5Rs
eTable 3. Distribution of Primary Cause of Severe Maternal Morbidity in Surveillance and Non-Surveillance Hospitals, 2019

This supplementary material has been provided by the authors to give readers additional information about their work.

© 2022 Wolfson C et al. *JAMA Network Open.*

eFigure 1. Severe Maternal Morbidity Surveillance Definition

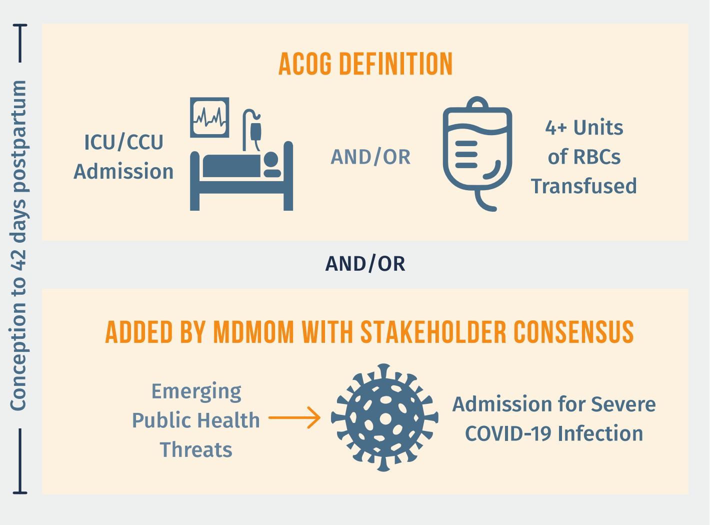

Notes: ICU, intensive care unit; CCU, critical care unit; RBC, red blood cell.

© 2022 Wolfson C et al. *JAMA Network Open.*

eFigure 2. Timing of Severe Maternal Morbidity Events

a. All SMM Events (N=192)

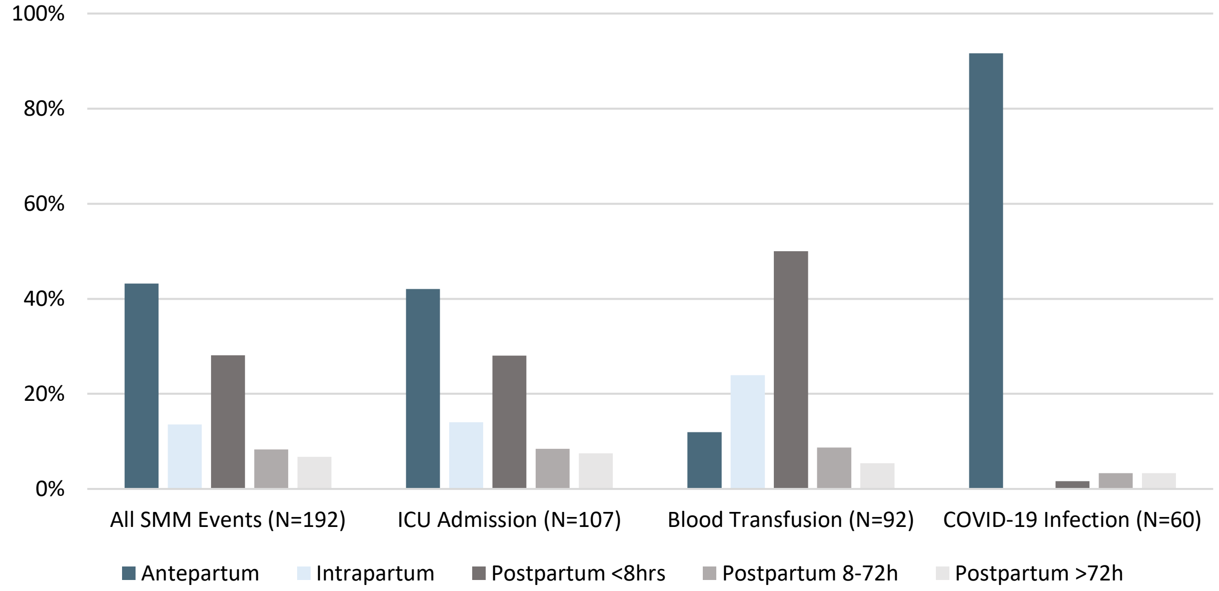

b. Excluding COVID-19 (N=134)

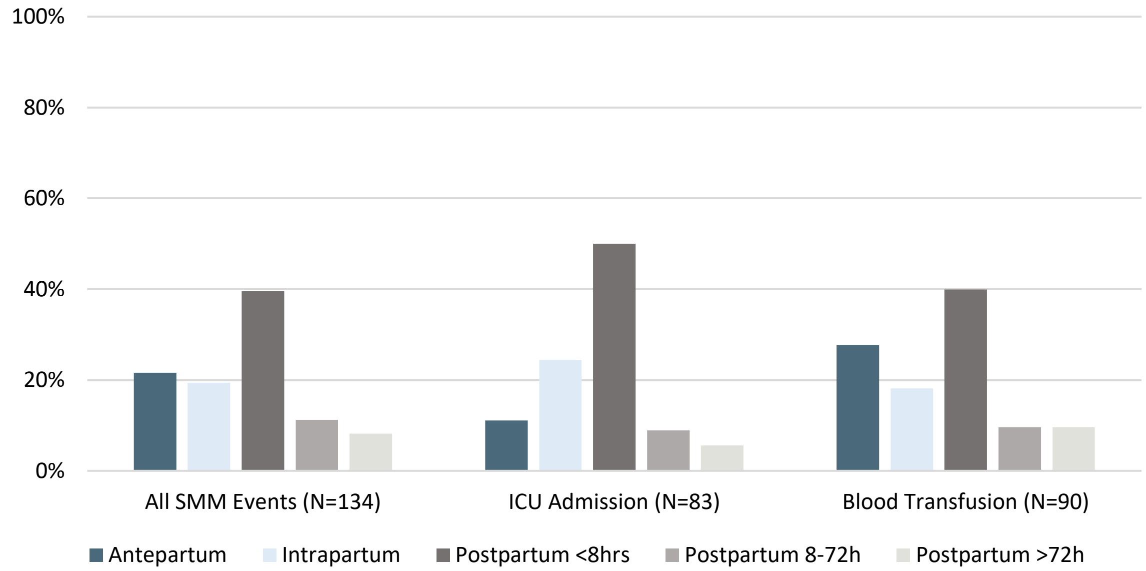

Notes: ICU, intensive care unit. SMM events include patients during pregnancy or within 42 days postpartum who are (a) admitted to an intensive care unit or critical care unit; and/or (b) with 4 or more units of red blood cells transfused; and/or (c) admitted to hospital for treatment of COVID-19 infection. Data source: Maryland SMM Surveillance and Review Database.

© 2022 Wolfson C et al. *JAMA Network Open.*

eFigure 3. Gestational Age at the Time of Antepartum and Intrapartum SMM Events

a. All SMM Events (N=192)

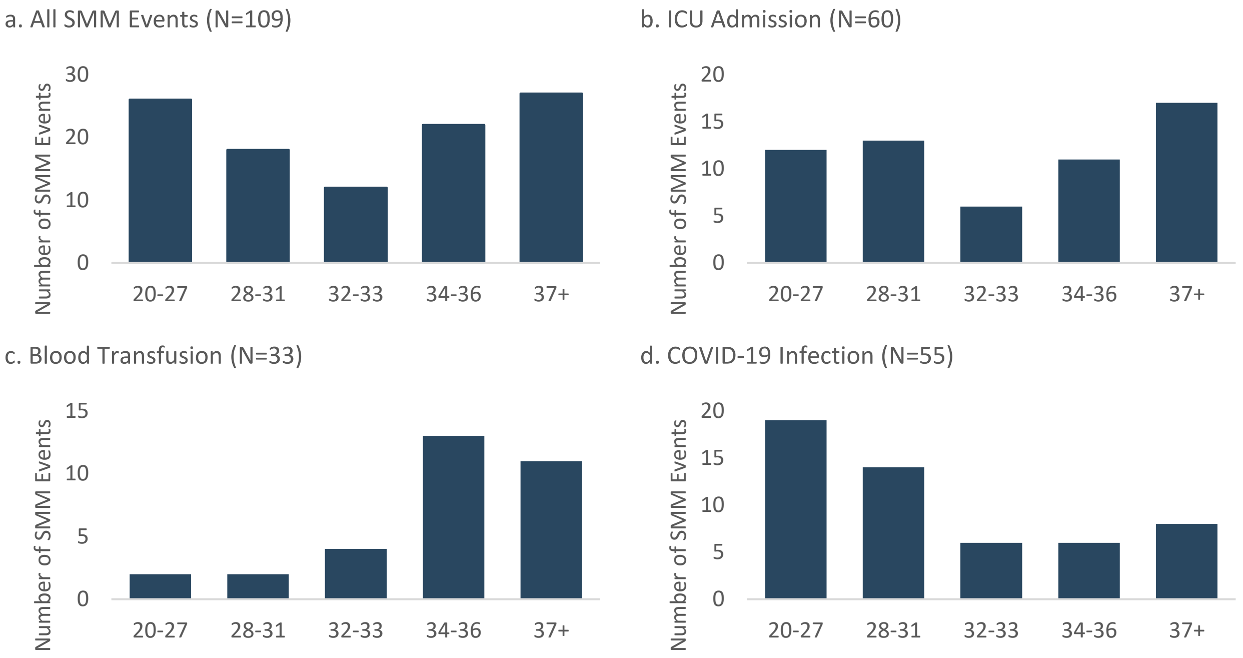

b. Excluding COVID-19 (N=134)

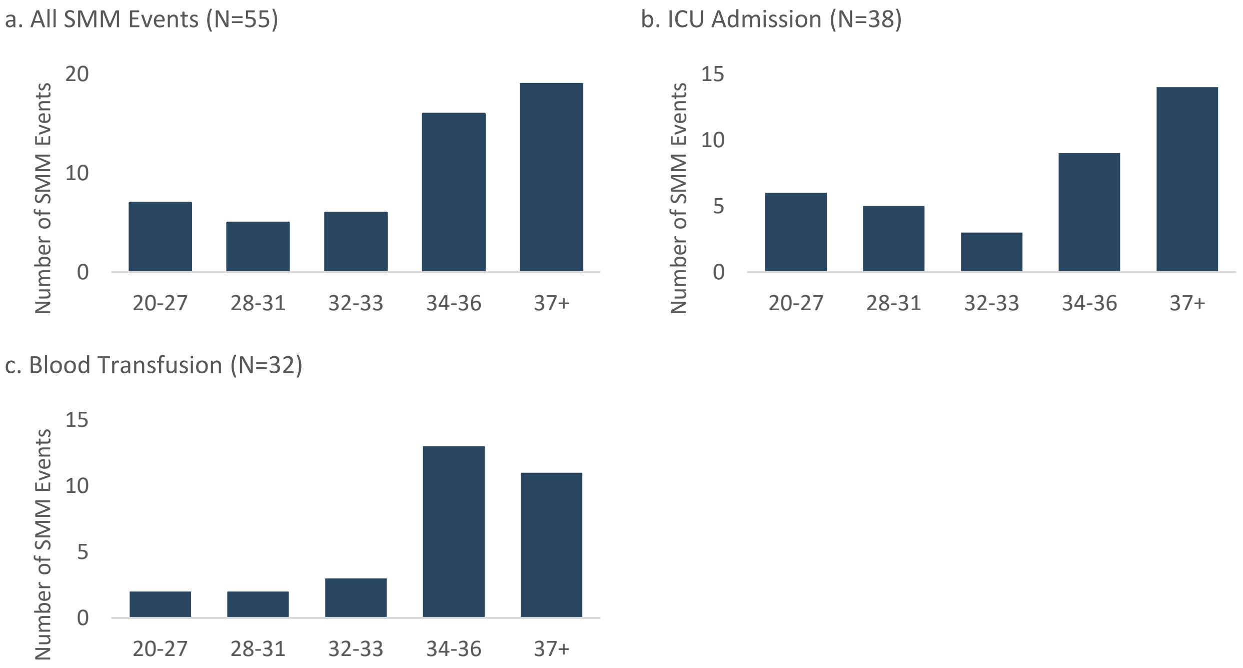

Notes: ICU, intensive care unit. Four SMM events occurred prior to 20 weeks' gestation. SMM events include patients during pregnancy or within 42 days postpartum who are (a) admitted to an intensive care unit or critical care unit; and/or (b) with 4 or more units of red blood cells transfused; and/or (c) admitted to hospital for treatment of COVID-19 infection.

Data source: Maryland SMM Surveillance and Review Database.

© 2022 Wolfson C et al. *JAMA Network Open.*

eTable 1. Blood Loss and Transfusion in Patients With SMM by Primary Cause

	All SMM Events (N=192)	Obstetric Hemorrhage (N=83)	COVID-19 Infection (N=57)	Hypertensive Disorder of Pregnancy (N=17)
Abnormal blood loss, n (%)	101 (53)	83 (100)	4 (7)	6 (35)
QBL, mean (SD)	3,360.1 (2,131.9)	3,587.6 (2,155.7)	1,816.8 (1,180.3)	4,091.5 (2,252.7)
Units of blood products transfused, mean (SD)	9.4 (6.6)	9.9 (6.7)	4.8 (5.0)	8.7 (6)
Units of blood products transfused, range	1-46	3-46	1-12	4-16
Massive Transfusion Protocol called, n (%)	34 (35)	33 (41)	0 (0)	1 (17)

Notes: QBL, quantitative blood loss in milliliters. QBL and units of blood products transfused missing for 3 patients with abnormal blood loss.
Data source: Maryland SMM Surveillance and Review Database.

© 2022 Wolfson C et al. *JAMA Network Open.*

eTable 2. Condensed Recommendations for Care Improvement Organized by the 5Rs

**Readiness: Ability to use an institution's resources, protocols and procedures when needed**	Enhance readiness for obstetric hemorrhage management
	Enhance readiness for blood transfusion
	Offer specific training to providers on the management of antepartum anemia
	Offer specific training to providers on the management of severe hypertension/preeclampsia
	Ensure availability of diagnostic services during nighttime hours and weekends
**Recognition: Assessment and measurement**	Timely assessment of, screening for, and diagnosis of complications
	Enhance vital sign monitoring during hospitalization
	Follow-up on abnormal test results
**Response: Treatment and management**	Timely initiation of treatment for patients with severe range blood pressure values
	Timely initiation of treatment for patients with abnormal bleeding
	Implement surgical care per clinical guidance
	Strengthen teamwork and communication within labor and delivery units
	Timely engagement with specialists
	Coordination of care within and across hospital systems and warm handoff of patient
**Reporting and System Learning: Communication, debrief, and review**	System learning to better manage obstetric hemorrhage events
	System learning to better manage blood transfusion events
	System learning to ensure safe anesthesia and surgical care
**Respectful Care: Recognizing the patient's right to be educated, informed, and supported**	Consider patients' individual circumstances when making discharge recommendations
	Implement implicit bias training across hospital departments

Data source: Maryland SMM Surveillance and Review Database.

© 2022 Wolfson C et al. *JAMA Network Open.*

eTable 3. Distribution of Primary Cause of Severe Maternal Morbidity in Surveillance and Non-Surveillance Hospitals, 2019

**Primary Cause of SMM**	**Non-Pilot (N=47,825)**		**Pilot (N=16,031)**	
	**No.**	**%**	**No.**	**%**
Hemorrhage	176	49.2	64	42.7
Respiratory conditions	11	3.1	7	4.7
Cardiac conditions	17	4.7	14	9.3
Renal conditions	48	13.4	16	10.7
Sepsis	43	12.0	11	7.3
Other obstetric	41	11.5	23	15.3
Other medical	22	6.1	15	10.0
			Pearson X^2	11.6
			P	0.072

Notes: Chi-square of difference in distribution of primary SMM cause between pilot and non-pilot hospital in 2019.
Data source: Maryland SMM Surveillance and Review Database; Agency for Healthcare Research and Quality's Maryland State Inpatient Databases during 2019.

© 2022 Wolfson C et al. *JAMA Network Open.*
